# Annexin‐A1: The culprit or the solution?

**DOI:** 10.1111/imm.13455

**Published:** 2022-03-01

**Authors:** Lauren Kelly, Sarah McGrath, Lewis Rodgers, Kathryn McCall, Aysin Tulunay Virlan, Fiona Dempsey, Scott Crichton, Carl S. Goodyear

**Affiliations:** ^1^ Institute of Infection, Immunity and Inflammation University of Glasgow Glasgow UK; ^2^ Medannex Ltd Fountainbridge, Edinburgh UK

**Keywords:** Annexin‐A1, biomarker, cancer, cell signalling inflammation, formyl peptide receptors, immune response

## Abstract

Annexin‐A1 has a well‐defined anti‐inflammatory role in the innate immune system, but its function in adaptive immunity remains controversial. This glucocorticoid‐induced protein has been implicated in a range of inflammatory conditions and cancers, as well as being found to be overexpressed on the T cells of patients with autoimmune disease. Moreover, the formyl peptide family of receptors, through which annexin‐A1 primarily signals, has also been implicated in these diseases. In contrast, treatment with recombinant annexin‐A1 peptides resulted in suppression of inflammatory processes in murine models of inflammation. This review will focus on what is currently known about annexin‐A1 in health and disease and discuss the potential of this protein as a biomarker and therapeutic target.

## Graphical abstract

Annexin‐A1 has a well‐defined anti‐inflammatory role in the innate immune system, but its function in adaptive immunity remains controversial. This glucocorticoid‐induced protein has been implicated in a range of inflammatory conditions and cancers, as well as being found to be overexpressed on the T cells of patients with autoimmune disease. This review will summarise what is currently known about ANXA1 in terms of its structure and function and discuss current research examining the role of ANXA1 and its receptors in disease. The potential of ANXA1 and its receptors as biomarkers and therapeutic targets in disease will also be discussed Special Issue: No.

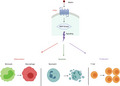

**Abbreviations**
AM: Alveolar macrophages
ANXA1: Annexin‐A1
aSAH: Aneurysmal subarachnoid haemorrhage
BAL: Bronchoalveolar lavage
COPD: Chronic obstructive pulmonary disease
DC: Dendritic cell
EAE: Experimental autoimmune encephalomyelitis
ERK: Extracellular signal‐related kinase
FPR: Formyl peptide receptor
GC: Glucocorticoid
GM‐CSF: Granulocyte‐macrophage colony‐stimulating factor
Hu‐r‐ANXA1: Human recombinant annexin‐A1
IPF: Idiopathic pulmonary fibrosis
LDLR: Low‐density lipoprotein receptor
MAPK: Mitogen‐activated protein kinase
MI: Myocardial infarction
MM: Multiple myeloma
MS: Multiple sclerosis
RA: Rheumatoid arthritis
RASF: Rheumatoid arthritis synovial fibroblast
SIV: Simian immunodeficiency virus
SLE: Systemic lupus erythematosus
TCR: T‐cell receptor
TNF: Tumour necrosis factor
WT: Wild type

## INTRODUCTION

Annexin‐A1 (ANXA1) is a member of the annexin superfamily of proteins that bind phospholipids in a calcium‐dependent manner. Annexins are capable of a range of biological functions, from structural organization of the cell to regulation of growth and vesicle trafficking [1]. More specifically, ANXA1 has established roles in both apoptosis, cell differentiation and modulation of the inflammatory response [2]. ANXA1 has been implicated in a range of diseases, playing both pro‐ [3] and anti‐inflammatory [4] roles in inflammatory conditions, as well as an involvement in cancer metastasis, invasiveness and proliferation [5, 6.

Under homeostatic conditions, ANXA1 is expressed mostly in the cytosol of innate immune cells such as monocytes, eosinophils and neutrophils. It is also expressed at lower levels by lymphocytes [7, 8. ANXA1 is well established as an anti‐inflammatory protein in the innate immune system; however, its role in adaptive immunity is less clear, perhaps partly due to its lower expression on adaptive immune cells [8].

Evidence has emerged to suggest that the anti‐inflammatory effects of ANXA1 are mediated by glucocorticoids (GCs), through both genomic and non‐genomic processes in both health and disease [9, 10, 11. GCs are a class of steroid hormones that function as inflammatory mediators and regulate a plethora of physiological processes. GCs have been adapted for use therapeutically in a wide range of diseases, but their beneficial effects can often be surpassed by side‐effects which range from increased infections to osteoporosis. Furthermore, long‐term usage of GCs can also result in tissue‐specific resistance to these therapies [12, 13, 14. This research has highlighted a potential molecular pathway involving ANXA1 that is activated by GCs to allow them to exert their pharmacological actions. This could perhaps be manipulated to minimize associated side‐effects and drug resistance. Therefore, further investigations into ANXA1 and how it functions are vital. This review will summarize what is currently known about ANXA1 in terms of its structure and function and discuss current research examining the role of ANXA1 and its receptors in disease. The potential of ANXA1 and its receptors as biomarkers and therapeutic targets in disease will also be discussed.

## ANXA1 IN IMMUNITY

### ANXA1 structure and externalization

ANXA1 is a 37‐kDa protein consisting of 346 amino acids. It is part of the annexin superfamily of proteins of which there are 12 members in vertebrates. Like other members of the annexin family, ANXA1 has a core region consisting of four repeating motifs containing type 2 calcium binding domains [15], and an N‐terminal domain which is unique to each member of the annexin family [8].

**FIGURE 1:**
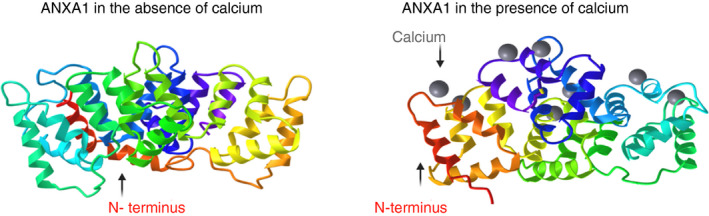
Annexin‐A1 alone and in the presence of calcium. Ribbon diagrams showing full‐length annexin‐A1 (ANXA1) without calcium binding (left, protein database code 1HM6) and with calcium binding (right, protein database code 1MCX). The N terminus is highlighted in red and calcium ions are in grey. The image shows a conformational change in the ANXA1 protein upon calcium binding (B), exposing the N terminus for subsequent binding to membrane phospholipids to facilitate its functions. The images were taken from the protein database using the iCn3D [150] software and edited using Biorender.com

In the presence of calcium, ANXA1 undergoes a critical conformational change which allows the core region to bind to membrane phospholipids and for the N‐terminal domain to be exposed allowing interaction with cellular receptors (Figure 1).

The N‐terminal domain mediates the anti‐inflammatory functions of ANXA1 including inhibition of leukocyte migration and the transendothelial passage of neutrophils [16, 17, 18. In contrast, the core region facilitates several functions such as membrane fusion and aggregation [19, 20 and has been shown to act in a pro‐inflammatory manner by promoting clustering and migration across the endothelium [3]. This suggests that the ‘opposite ends’ of ANXA1 play contrasting roles in an inflammatory setting.

To exert its biological effects, ANXA1 needs to be externalized from the cell it is contained within. Post‐translational modification of the ANXA1 protein, specifically phosphorylation at serine‐27, is essential for secretion of the protein. This was evident in experiments in which ANXA1 without this modification could not be released [15, 21. When the cell is activated during an inflammatory response, ANXA1 is translocated to the cell membrane where it is secreted, allowing it to interact with its receptors in an autocrine, paracrine or juxtacrine manner. Notably, ANXA1 expression is highly regulated by GCs and it is believed that the anti‐inflammatory effects of these molecules are mediated by ANXA1 [8, 22.

#### ANXA1 receptors

ANXA1 is thought to mediate the majority of its effects through formyl peptide receptors (FPRs). The FPRs are a family of G protein‐coupled receptors of which there are three in humans: FPR1, FPR2 and FPR3 [8, 23 These receptors bind a wide range of ligands, including those involved in chemotaxis and activation of phagocytes [24].

**FIGURE 2:**
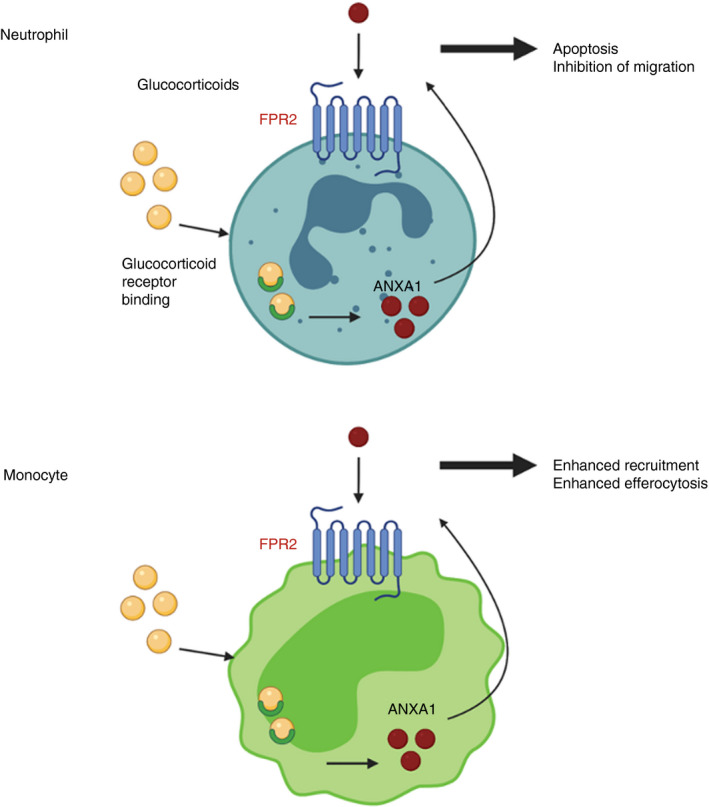
Actions of FPR2 are cell type dependent. Internalization and binding of glucocorticoids (GCs) to their receptor triggers the production of ANXA1. ANXA1 is externalized where it can bind to FPR2 on the cell surface initiating functions such as apoptosis and inhibition of migration in neutrophils and enhanced recruitment and efferocytosis in monocytes. Image created with Biorender.com

Neutrophils can mediate both pro‐inflammatory and anti‐inflammatory functions via FPRs. The most versatile of these receptors is FPR2, which can interact with a variety of ligands resulting in diverse pro‐inflammatory (cathelicidin) and anti‐inflammatory (lipoxin A4) effects depending on the particular ligand bound [25] (Figure 2).

**FIGURE 3:**
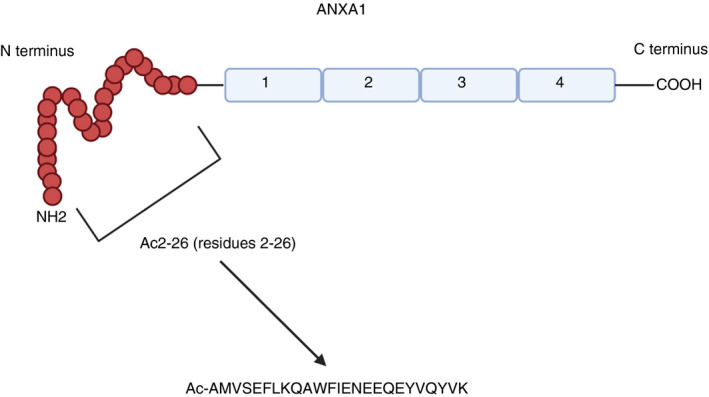
ANXA1 structure. ANXA1 is composed of four repeating C terminal motifs and an N terminal domain consisting of the Ac2‐26 peptide. Both full‐length ANXA1 and Ac2‐26 have been shown to interreact with the FPRs. The sequence of Ac‐26 is shown, along with its acetyl group N terminus constructed for stability and delay of protein degradation. Image adapted from Gavins et al [8] and created using Biorender.com

ANXA1 has been shown to interact primarily with FPR1 and FPR2 in the context of many diseases. Interestingly, N‐terminal peptides, including Ac2‐26 (Figure 3) as well as the full‐length ANXA1 protein, have been shown to activate the FPR family of receptors [26, 27, 28. Most of the interactions mediated by the FPR receptors seemed to be involved in host clearance and tissue maintenance processes.

The interaction between ANXA1 and FPR1 has been suggested to result in anti‐inflammatory processes in diseases such as cancer, where interactions of these two proteins on murine dendritic cells (DCs) were shown to establish DC/corpse synapses and clearance of dead cells [29]. Contrastingly, studies have suggested that ANXA1‐FPR1 interactions can drive oncogenic processes including cellular proliferation [30] and invasion [5]. Human studies in an inflammatory setting (cutaneous adverse drug reactions) suggest that the FPR1‐ANXA1 interaction is involved in mediating necroptosis [31], again suggesting a pro‐inflammatory role.

In the case of FPR2, exposure of neutrophils and monocytes to GCs has been shown to increase the expression of this receptor, suggesting it is playing a role in mediating anti‐inflammatory interactions [32]. Furthermore, antibody‐mediated blocking of FPR2 prevented anti‐inflammatory functions mediated by ANXA1, such as inhibition of neutrophil transmigration [11]. Interestingly, ANXA‐FPR2 interactions have been associated with a transient increase in the levels of active alveolar macrophages (AM), which are further associated with anti‐viral functions. An increased expression of the AM regulating cytokine, granulocyte‐macrophage colony‐stimulating factor (GM‐CSF), was also observed. These effects were decreased upon addition of an FPR2 antagonist, but remained upregulated after genetic deletion of FPR1, suggesting this anti‐viral function could not be conveyed via FPR1. This supports the idea of differing functions of these two receptors [33] and leads to the hypothesis that depending on the environment (e.g. viral infection vs chronic inflammatory state), ANXA1 signals through either FPR1 or FPR2 to induce pathways required for resolution. Fully understanding this biology could be beneficial in terms of providing a more specific target for anti‐viral and also anti‐inflammatory therapies. The anti‐viral aspect of this is particularly relevant during the current COVID‐19 pandemic, where there is an immense unmet need for alternative targets to help dampen the hyperinflammatory state observed as a consequence of this viral disease.

### ANXA1 in the innate immune system

The innate immune system is widely referred to as the human body's first line of defence against invading pathogens. It consists of several physical, chemical and microbiological barriers, as well as cellular components, all working cohesively to protect the body.

The innate immune response depends on several cell types, including monocytes, dendritic cells and neutrophils: each responsible for particular functions. The resulting effect is the production of a plethora of cytokines and chemokines, further activating additional cells to help clear the pathogen [34].

As sustained inflammation can be damaging, it is important that once a pathogen is cleared, the inflammatory response is resolved rapidly [35]. This is controlled by several anti‐inflammatory mediators, one of which is suggested to be ANXA1 [23]. During homeostasis, granulocytes and monocytes of the innate immune system contain vast amounts of intracellular ANXA1 within the cytoplasm. When an inflammatory response occurs, ANXA1 is transported to the cell surface where it binds to the plasma membrane in a calcium‐dependent manner [36]. Once activated, ANXA1 exerts anti‐inflammatory effects such as inhibition of neutrophil and monocyte adhesion to the endothelium. Mechanistically, this is thought to be due to ANXA1 competing with the endothelial integrin counterreceptor, VCAM‐1 for binding to the adhesion molecule α_4_β_1_ integrin [37, 38.

The role of ANXA1 and its modulation of the inflammatory response has also been demonstrated in studies using ANXA1 knockout (‐/‐) mice. In addition to the enhanced inflammatory responses observed in these knockout mice, monocytes and neutrophils were more sensitive to activation. Moreover, loss of ANXA1 rendered mice more unresponsive to the effects of GCs. Notably, it was harder to dampen the innate immune cell‐mediated inflammatory response. Combined, these data provided evidence that the ability of ANXA1 to reduce innate immune cell‐mediated inflammation is GC dependent [39, 40. This was further supported in human studies that demonstrated higher expression of ANXA1 in alveolar macrophages obtained from bronchoalveolar lavage (BAL) samples of patients with asthma and interstitial lung disease that had received GCs [41].

### ANXA1 in the adaptive immune system

The adaptive immune response is a long‐lived, antigen‐specific response involving T cells and B cells [8]. Cells of the adaptive immune system have the ability to recognize a wide range of specific pathogenic antigens upon re‐exposure. This allows them to react in an amplified, rapid fashion to prevent significant re‐infection and subsequent damage to the body [42].

The role of ANXA1 in adaptive immunity is far from understood, perhaps due to its lower expression on adaptive immune cells. Inactivated T cells have been shown to express little to no ANXA1 and FPR2; however, their expression is increased upon activation of these cells. CD4^+^ T cells have been shown to express more ANXA1 than CD8^+^ T cells, with CD4^+^ memory cells expressing more ANXA1 than naïve CD4^+^ T cells [7, 8, 43. Studies have shown that murine naive T cells cultured in the presence of human recombinant ANXA1 (h‐r‐ANXA1) produced more Th1‐associated transcription factors (such as T‐bet) compared to control cells that were not cultured in the presence of h‐r‐ANXA1. These cells also produced increased amounts of Th1‐associated cytokines such as IL‐2 and decreased amounts of IL‐4, a cytokine associated with decreased Th1 production. This was enhanced in Th1 skewing conditions indicating that ANXA1 might have a regulatory effect on cytokines that modulate this skewing. It also adds evidence to the idea that ANXA1 could perhaps play a role in the early stages of determination of T‐cell differentiation [43, 44, 45. D’Acquisto *et al*. have further speculated that ANXA1 may influence this by altering the strength of T‐cell receptor (TCR) signalling [44].

**FIGURE 4:**
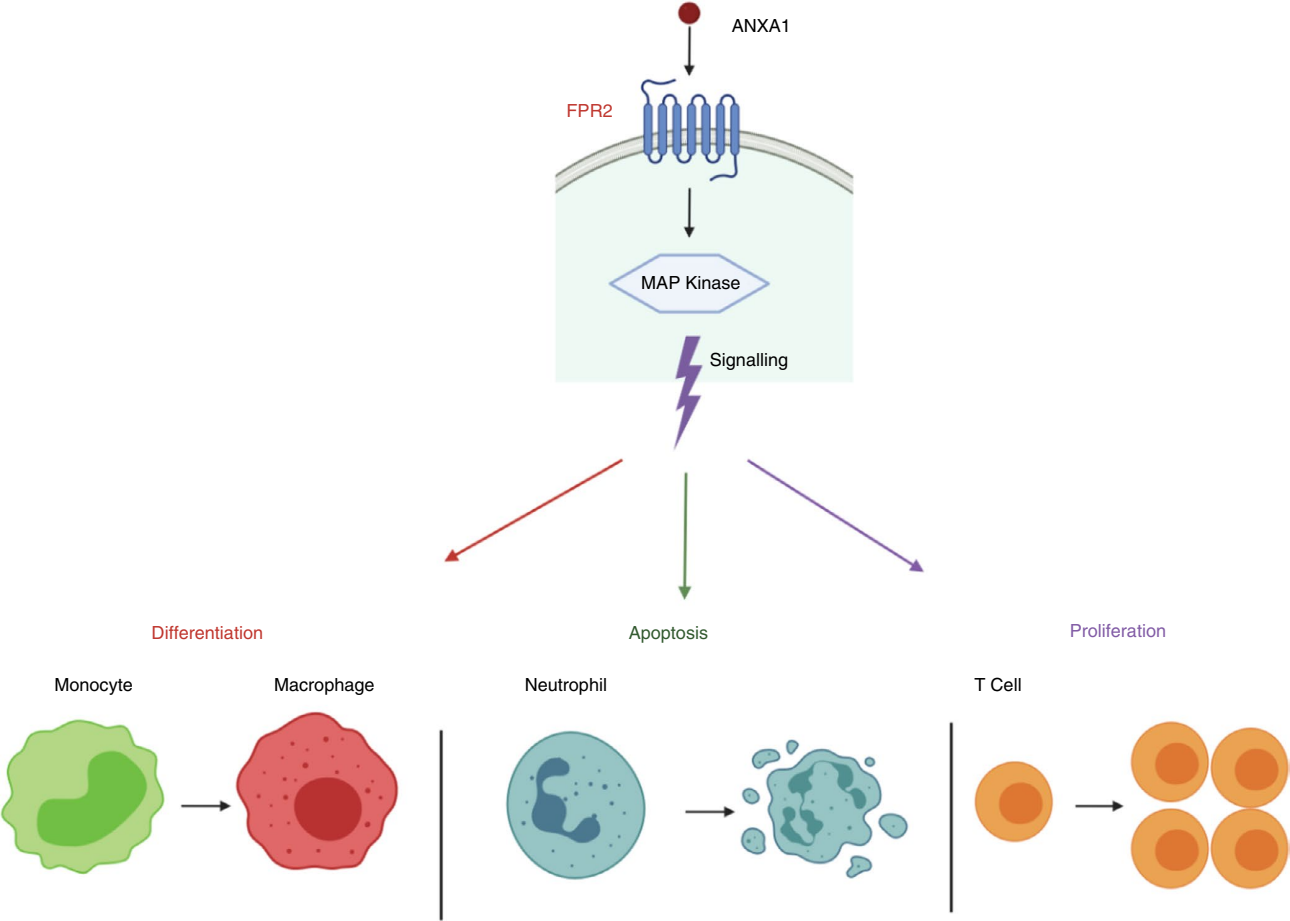
ANXA1 Triggers MAP kinase signalling. ANXA1 binding to FPR2 on the plasma membrane can trigger signalling of MAP kinases. This has different outcomes depending on the cell type affected, including monocyte differentiation, neutrophil apoptosis and T‐cell proliferation. Adapted from D’Acquisto et al [48] Image created with Biorender.com

ANXA1 has also been shown to be expressed on B cells. Interestingly, ANXA1 was observed to primarily reside on the surface of B cells, the opposite to what is seen in other immune cells, which could suggest differing functions for this protein even within adaptive immune cell types [7, 8, 46. Indeed, ANXA1 is known to be involved in signalling pathways that, once induced, can have different implications dependent on the cell type. For example, ANXA1 is known to activate signalling pathways involving mitogen‐activated protein kinases (MAPK), which can lead to monocyte differentiation, T‐cell proliferation and neutrophil apoptosis [47, 48. This highlights the complex and differing functions of this protein within different cell types (Figure 4).

Several studies have suggested that ANXA1 plays an anti‐inflammatory role in adaptive immunity. The addition of purified ANXA1 to thymocytes resulted in loss of their suppressor T‐cell activity [49]. ANXA1 deficiency was also shown to enhance antigen‐dependent T‐cell proliferation and subsequent inflammation [50]. Furthermore, ANXA1 peptides were shown to inhibit cytokine production and antigen‐mediated cellular proliferation in atopic sensitized patient samples [51]. Combined, these studies insinuate that in states of uncontrolled inflammation, ANXA1 could potentially be released to drive anti‐inflammatory pathways. Indeed, other proteins act in this way, such as the metabolite itaconate, an anti‐inflammatory protein produced during an inflammatory macrophage response. Itaconate interestingly has been shown to modify ANXA1 during an inflammatory response to allow it to carry out anti‐inflammatory functions. It remains to be seen, however, whether these modifications do or do not occur in certain inflammatory diseases where modification is essential for ANXA1 to play its protective role [52].

In contrast, there is also evidence for a pro‐inflammatory role of ANXA1 in the adaptive immune system. D’Acquisto et al. investigated the effect of adding hu‐r‐ANXA1 to activated T cells *in vitro* and found that addition of this protein increased T‐cell proliferation and activation. This effect was only seen when the T cells were stimulated in conjunction with FPR2 receptor externalization, suggesting ANXA1 could mediate these pro‐inflammatory effects via FPR2 receptor. Furthermore, naïve T cells differentiated in the presence of hu‐r‐ANXA1, and increased skewing towards a Th1 phenotype, associated with more pro‐inflammatory activity [15, 43.

## ANXA1 IN DISEASE

The above sections have primarily focused on the role of ANXA1 in the normal physiology, ranging from modulation of inflammation to regulation of proliferation. However, there is an increasing evidence that ANXA1 can play a role in disease, including inflammatory disease and cancer progression (Table 1). Similar to findings in health, the role of ANXA1 in disease is not entirely clear, with alternating roles being discussed within the same disease, as well as within different disease subsets.

**TABLE 1:** Expression of ANXA1 in different disease settings

Disease	ANXA1 Expression	Reference
Systemic Lupus Erythematosus	Increased	Bruschi, M. et al. Annexin a1 and autoimmunity: From basic science to clinical applications. International Journal of Molecular Sciences. **19** (2018).
Sepsis	Decreased	Tsai, W. H., Shih, C. H., Yu, Y. Bin & Hsu, H. C. Plasma levels in sepsis patients of annexin A1, lipoxin A4, macrophage inflammatory protein−3a, and neutrophil gelatinase‐associated lipocalin. J. Chinese Med. Assoc. **76,** 486–490 (2013).
Rheumatoid Arthritis	Increased	D’acquisto, F. et al. Glucocorticoid treatment inhibits annexin−1 expression in rheumatoid arthritis CD4+ T cells. *Rheumatology* **47**, 636–639 (2008)
Idiopathic Pulmonary Fibrosis	Increased autoantibodies	Bringardner, B. D., Baran, C. P., Eubank, T. D. & Marsh, C. B. The role of inflammation in the pathogenesis of idiopathic pulmonary fibrosis. Antioxidants and Redox Signaling **10**, 287–301 (2008).
Multiple Sclerosis	Decreased	Colamatteo, A. et al. Reduced Annexin A1 Expression Associates with Disease Severity and Inflammation in Multiple Sclerosis Patients. J. Immunol. **203**, 1753–1765 (2019).
Lung cancer	Increased	Biaoxue, R. et al. Upregulation of Hsp90‐beta and annexin A1 correlates with poor survival and lymphatic metastasis in lung cancer patients. J. Exp. Clin. Cancer Res. **31**, 1–14 (2012).
Pancreatic cancer	Increased	Oliveira‐Cunha, M., Byers, R. J. & Siriwardena, A. K. Poly(A) RT‐PCR measurement of diagnostic genes in pancreatic juice in pancreatic cancer. Br. J. Cancer **104**, 514–519 (2011).
Prostate cancer	Decreased	Kang, J. S. et al. Dysregulation of annexin I protein expression in high‐grade prostatic intraepithelial neoplasia and prostate cancer. Clin. Cancer Res. **8,** 117–123 (2002).
Laryngeal cancer	Decreased	Silistino‐Souza, R. et al. Annexin 1: Differential expression in tumor and mast cells in human larynx cancer. Int. J. Cancer **120**, 2582–2589 (2007).
Breast cancer	Increased	Graauw, M. de et al. Annexin A1 regulates TGF‐β signaling and promotes metastasis formation of basal‐like breast cancer cells. Proc. Natl. Acad. Sci. U. S. A. **107**, 6340 (2010).

### ANXA1 and the innate immune response in disease

Evidence for a role of ANXA1 in modulating the activity of innate inflammatory cells has also been explored. For instance, in multiple sclerosis (MS), increased expression of ANXA1 has been observed in macrophages at the sites of active lesions [53]. In contrast, reduced ANXA1 levels have been found in brain parenchymal capillaries within MS patients. However, this fall in ANXA1 expression occurred at sites distant from active lesions [54].

In models of myocardial infarction (MI), treatment with exogenous Ac2‐26 at the onset of reperfusion improved recovery of left ventricle function in rat and murine hearts, as well as preventing cardiomyocyte damage. The protective effects of Ac2‐26 were subsequently diminished upon addition of an FPR1 antagonist. Interestingly, the addition of a FPR2 antagonist only modestly reduced cardio‐protection and this was short‐lived compared to the FPR1 antagonist [55]. This could suggest that the protective role of ANXA1 in MI is mostly mediated through FPR1 rather than FPR2. Studies in patients with carotid stenosis further support this protective role of ANXA1, as higher ANXA1 gene [56] and protein [57] expression was seen in patients who were asymptomatic compared with those exhibiting symptoms.

Atherosclerosis is another disease mediated by innate immune cell involvement, and a protective role for ANXA1 in modulating this disease has been widely explored. Atherosclerosis is a well‐recognized contributor to cardiovascular disease and is characterized by formation of plaques in the artery walls, leading artery narrowing and reduced blood flow. It is known to be triggered by high levels of cholesterol in the blood, leading to lipoprotein retention in the artery walls, which triggers inflammation. A continuation of this atherosclerotic process can eventually progress to MI or stroke [58, 59.

Macrophages are key pro‐resolving players in the inflammatory process that occurs within atherosclerotic plaques via their ability to remove lipoproteins. This function becomes dysregulated with continued leakage of lipids from the blood vessels, leading to macrophages becoming inflammatory ‘foam’ cells, which are packed with lipids. Researchers have therefore claimed that recruitment of leukocytes such as macrophages is a key process in the progression of atherosclerosis [60].

A protective role has been implicated for ANXA1 in atherosclerosis in a study showing that the ANXA1 synthetic N‐terminal peptide, Ac2‐26, is able to reduce neutrophil and monocyte‐mediated atherosclerotic plaque formation [61]. These protective effects are also seen in combination with the ANXA1 receptors. Administration of Ac2‐26 in mice reduced recruitment of myeloid cells, an effect that was shown to be mediated through FPR2. Repeated administration of Ac2‐26 was shown to reduce the size of atherosclerotic lesions and accumulation of macrophages in these lesions [62]. Studies in the low‐density lipoprotein receptor (LDLR)^−/−^ mouse model of atherosclerosis support this protective role and show that administration of hu‐r‐ANXA1 to mice fed a western diet attenuated plaque progression. Results also showed that hu‐r‐ANXA1 could mediate FPR2‐dependent neutrophil rolling, reducing the plaque inflammation [63].

De Jong et al have highlighted the importance of the role of ANXA1 in leukocyte recruitment and macrophage polarization, which are key processes that are known to be dysregulated in atherosclerosis [58]. The group also suggested the role of ANXA1 as an anti‐inflammatory mediator in cardiovascular therapeutics should not be overlooked and have highlighted several studies that show the benefits of ANXA1 and its synthetic peptide in atherosclerosis [64], stroke [65] and MI [66].

### ANXA1 and the adaptive immune response in disease

In addition to studies that are investigating the role of ANXA1 in the disease associated‐innate immune compartment, there is also a focus on the adaptive immune system. Immune cells involved in the adaptive immune response that have received particular attention, especially with regard to ANXA1, are T cells. These are described in the literature as being key players in autoimmune and inflammatory disease [67].

A range of autoimmune inflammatory‐mediated diseases including rheumatoid arthritis (RA), and multiple sclerosis (MS) are known to be associated with T‐cell‐mediated pathology [67]. However, what causes the dysregulated activity of these T cells is currently unknown. Several groups have reported an increased level of ANXA1 in an inflammatory setting, in both animal models of disease [68, 69 and in human samples [70]. Experimental autoimmune encephalomyelitis (EAE) mouse models of inflammation have shown that ANXA1 levels correlated with disease severity. Moreover, ANXA1^−/−^ mice showed reduced severity of disease upon induction of EAE compared to WT mice [71]. CD4^+^ T cells in patients with RA have been shown to have increased ANXA1 expression in comparison to healthy controls [43]. In addition, GC treatment for this disease has been shown to reduce ANXA1 expression in CD4^+^ T cells in RA patients in a time‐ and concentration‐dependent manner [72] perhaps contradicting the hypothesis that ANXA1 is a downstream mediator of GCs. ANXA1 has also been found to be released from RA synovial fibroblasts (RASF) following tumour necrosis factor (TNF)‐a‐mediated activation, and to promote RASF matrix metalloproteinase‐1 secretion [73], known to play a major role in collagen degradation.

One hypothesis suggests that ANXA1 could function as an antigen in inflammatory diseases, which triggers the production of destructive autoantibodies rather than the protective ones [48]. A study which supports this idea showed that in patients with idiopathic pulmonary fibrosis (IPF), ANXA1 was associated with increased CD4^+^ T‐cell activity, as well as autoantibody production in patients with exacerbated IPF [74]. Raised levels of ANXA1 autoantibodies have also been seen in patients with inflammatory bowel disease [75] and systemic lupus erythematosus (SLE), particularly in lupus nephritis [76, 77, indicating a potential role for ANXA1 on B cells in these diseases. However, further work is needed to determine a role for these autoantibodies in the pathogenesis of these diseases.

Other studies have shown ANXA1 to be anti‐inflammatory in murine models of autoimmune disease. In models of contact hypersensitivity, collagen‐induced arthritis and inflammation induced by transgenic T cells, deficiency of ANXA1 was associated with exacerbated inflammation. In particular, loss of ANXA1 in a model of arthritis was associated with increased antigen‐specific T‐cell activation [78]. However, as there are limited studies to support this anti‐inflammatory role of ANXA1 in T‐cell‐associated disease, it is evident further data are needed to make this conclusion.

### ANXA1 in Th17‐associated diseases

Th17 cells are a subtype of IL‐17‐secreting CD4^+^ T cells that are primarily pro‐inflammatory and normally play a role in the body's defence against bacterial and fungal infections [79, 80. However, uncontrolled activation of Th17 cells has been shown to be a hallmark of several autoimmune and inflammatory diseases [81, 82, 83. We have seen evidence for a role for ANXA1 in mediating T‐cell pathology in autoimmune disease, so could this protein be involved in manipulating Th17 activity? Unfortunately, there is limited research regarding the role of ANXA1 in Th17‐mediated diseases, and the available data in the area are conflicting.

In a murine model of autoimmune uveitis, ANXA1‐/‐ mice had enhanced retinal inflammation associated with overactivation and proliferation of Th17 cells. Furthermore, the addition of hu‐r‐ANXA1 reduced inflammation and inflammatory cytokine production, including IL‐17 [84]. Murine models of atopic dermatitis also demonstrated that knocking out ANXA1 upregulated IL‐17A [85]. In patients with chronic inflammation (e.g. MS), Th17 cells exhibited a significant reduction in ANXA1 production, also reflecting how downregulation of this protein is associated with increased inflammation [86]. Th17 responses have also been shown to negatively correlate with ANXA1 expression in other models of inflammation, including in studies in primates with simian immunodeficiency virus (SIV) infection [87].

In contrast, an ANXA1^−/−^ mouse model of (MOG_35‐55_ induced) EAE exhibited a reduced Th17 profile and had reduced IL‐17 production in comparison to WT mice [71]. It has been suggested that the (MOG_35‐55_ induced) EAE model relies on early infiltration of Th1 cells for full induction of the disease. Interestingly, T cells from ANXA1^−/−^ mice had an impaired production of Th1‐associated cytokines, supporting the idea that these mice have a reduced ability to develop this model of EAE. This also suggests a potential pro‐inflammatory role for ANXA1 in modulating Th1‐mediated EAE disease pathology.

Transgenic mouse models generated to overexpress ANXA1 in T cells had an exacerbated inflammatory response (increased IL‐2 production upon stimulation) and worse clinical disease scores. Furthermore, accumulation of Th17 cells was evident in inflamed tissue [88].

These contrasting findings, alongside the fact that the majority of the data is from animal models, indicate that more research is required to fully understand the role of this protein in disease. More specifically, further analysis of patient and healthy control samples is needed to fully understand the role of ANXA1 in human disease. Often there are limitations with translating animal model data to the human disease, and these models do not always mimic the human disease in an accurate way. So, although mice models are invaluable to deepen understanding of disease mechanisms within a living system, caution should be taken when interpreting the data obtained from them.

### ANXA1 in cancer

Cancer is commonly described as being initiated through a series of mutations that result in the induction of abnormal rates of processes such as growth or angiogenesis [89]. A vital step in tumour growth is the ability of cancer cells to develop different ways of mimicking immune tolerance mechanisms to avoid detection from the immune system. Interestingly, chronic inflammation seems to play a key role in cancer progression by inducing stimulation of cancer cell proliferation and metastasis [90].

Similar to inflammatory disease, there is a large amount of literature suggesting a role for ANXA1 in cancer, but this role has not been well defined. ANXA1 has been shown to be overexpressed in cancers such as lung [91], pancreatic [92] and a number of breast cancer subtypes [93, 94, but contrastingly expressed at lower levels in other types of cancer such as prostate [95] and laryngeal [96, 97. It remains to be determined whether ANXA1 plays a dominant role in certain cancers. It is clear that expression of ANXA1 between different cancers is variable. Suggested mechanisms for this include mutations or deletions in the ANXA1 gene and epigenetic changes such as silencing of the ANXA1 promotor. Studies have also suggested that ANXA1 may be specific to each tumour type due to post‐translational modifications of the protein [97]. Indeed, post‐translational phosphorylation is required for transfer of ANXA1 across the membrane [21], which could account for alterations in surface expression seen on different cells and in different cancers.

Studies investigating the role of ANXA1 in tumour progression demonstrated that knocking out ANXA1 resulted in reduced tumour growth, angiogenesis and metastasis [98]. Furthermore, treatment of rats with an anti‐ANXA1 radioimmunotherapy destroyed tumours and increased survival in these animals [99]. It has also been suggested that ANXA1 has a role in the development of drug resistance [100] and the initiation of DNA repair [101]. These multiple functions make ANXA1 an important target within many cancers and emphasize the need for increased understanding of the exact mechanisms of ANXA1‐mediated cellular activation. This is particularly imperative in certain malignancies, such as breast cancer, where it has been shown that ANXA1 can behave as both a tumour suppressor (e.g. in oestrogen receptor^+^ breast cancers that generally have low ANXA1 levels) and an oncogene (e.g. in basal‐like breast cancer where ANXA1 is highly expressed), with levels of expression dependent on tissue and cell type [93, 94.

ANXA1 has also been shown to play a role cancer progression through interactions with immune cells. In particular, ANXA1 was associated with worst outcomes in cancer patients by enhancing regulatory T‐cell functions [102], promoting mast cell infiltration [103] and promoting polarization and activation of tumour‐associated macrophages [104]. Moreover, ANXA1 was shown to be a key component of extracellular vesicles released by pancreatic cancer cells and was able to influence the tumour microenvironment by triggering mesenchymal switches and cell motility on fibroblasts and endothelial cells. The mechanism of this was also shown to be mediated through the FPRs [105].

The ANXA1 receptors, FPRs, have also been implicated in several cancer types such as prostate and breast cancers [106, 107. Interaction of ANXA1 and these receptors has been shown to activate oncogenic pathways, such as those involving extracellular signal‐related kinase (ERK) phosphorylation resulting in activation of cell invasion. ERK pathway activation has been implicated in several cancer types, perhaps explaining the vast amount of literature suggesting a role for ANXA1 and its receptors in numerous different cancer types [5, 47.

### ANXA1 and the FPRs as potential biomarkers in disease

ANXA1 has been suggested as a potential biomarker for several diseases [97, 108. Recent data have emerged suggesting that ANXA1 could be a novel biomarker of congestion in acute cardiac failure [109]. Interestingly, an increased level of circulating ANXA1 been suggested as a biomarker for several inflammatory diseases. This has been observed in SLE where patients that present with renal complications have higher levels of circulating ANXA1 [110]. In addition, it has also been suggested that ANXA1 is a biomarker of glomerular injury [111]. Furthermore, increased serum ANXA1 has been identified in patients with chronic obstructive pulmonary disease (COPD) compared to healthy controls. High serum ANXA1 levels were associated with disease severity, with ANXA1‐induced fibroblast activation in the lungs being suggested as a potential mechanism [112]. However, these data should be interpreted carefully as changes in the circulating concentration of a protein do not necessarily mean a role in disease pathogenesis.

In contrast, plasma ANXA1 was shown to be decreased in sepsis patients compared to healthy controls [113]. Recent data have also provided evidence that serum ANXA1 levels could predict disease severity in a form of traumatic brain injury called aneurysmal subarachnoid haemorrhage (aSAH). Serum ANXA1 levels were significantly lower in patients with this condition than in healthy controls. Moreover, serum ANXA1 levels predicted a poor outcome 6 months after aSAH occurance [114]. Interestingly, a recent COVID‐19 study has shown that serum ANXA1 levels were significantly lower in samples from patients with severe disease compared to both healthy controls and those with moderate disease [115]. This suggests a potential role of ANXA1 as a biomarker in predicting COVID‐19 prognosis, which could allow for earlier treatment interventions. Researchers have further proposed the idea Ac2‐26 as an anti‐inflammatory mediator in treating patients with severe COVID‐19 but have agreed that further investigations are needed to determine the efficacy and safety of this [116].

Research has also suggested ANXA1 acts as a marker of sensitivity to endogenous GCs, which may account for the altered susceptibility to inflammatory diseases that are associated with dysregulation of these molecules [10]. In terms of looking at ANXA1 as a potential biomarker in T‐cell‐mediated diseases, there is a wide range of data suggesting measuring increased levels of protein could serve as a potential biomarker [43, 50, 70. However, substantially more human studies need to be done to be able to verify these findings.

Evidence has also indicated that ANXA1 could be a useful biomarker in certain forms of cancer. As ANXA1 seems to be expressed differently across a range of cancers, and this expression also seems to vary depending on factors such as tumour stage and metastasis [117], the idea of developing this protein as a biomarker for all cancers could be seen as slightly challenging. However, a few studies have provided data to support its use as a biomarker. ANXA1 has been suggested as a biomarker in cholangiocarcinoma [118] and several other cancers including colorectal [119], lung [120], liver [121], pancreatic [122] and breast cancers [123], all of which are extremely heterogeneous and require good biomarkers to inform treatment decisions. Interestingly, ANXA1 overexpression in epithelial ovarian cancer was shown to be a marker of better overall survival and it has been suggested that this may prove useful in determining what treatment strategies are put in place [124].

In terms of the FPRs, some studies have shown that FPR1 could be promising as a biomarker, for acute myocardial infarction [125], tuberculosis [126] and particularly in cancer [127, 128. Recent data have also suggested that a particular mutation in FPR1 is a good predictor of poor prognosis in advanced rectal cancer [129]. Likewise, most of the available data have evidenced a role for FPR2 as a marker of poor prognosis in cancer, particularly gastric cancer [130] and also epithelial ovarian cancer [131]. This is in contrast to the suggested role of ANXA1 in this form of ovarian cancer, suggesting the signalling of ANXA1 in this process may not be predominantly mediated through FPR2. However, further studies are needed to validate these findings.

#### ANXA1 and the FPRs as potential therapeutic targets

Hu‐r‐ANXA1 and the N‐terminal peptide of the protein, Ac2‐26, have both been tested as potential treatments in mice models of inflammation [64, 132. Treatment of murine neuroblastoma cells with hu‐r‐ANXA1 reduced the enzymatic degradation of the amyloid‐β (Aβ) protein associated with Alzheimer's disease pathogenesis. Addition of this protein to murine microglial cells also reduced the Aβ‐induced expression levels of cytokines such as TNF‐α and IL‐4, which are associated with the inflammatory process [133]. Furthermore, the addition of hu‐r‐ANXA1 to macrophages from murine models of liver inflammatory disease reduced their pro‐inflammatory responses [134]. There is very little evidence in the literature for a role for ANXA1 in B cells to date; however, experiments from Mihaylova et al in mice models of SLE have provided proof that ANXA1 could be a potential therapeutic target on these cells. Data showed that SLE mice treated with an anti‐ANXA1 antibody resulted in the inhibition of both T‐cell activation and proliferation compared to control groups [135]. Furthermore, treatment with the anti‐ANXA1 antibody was associated with prolonged survival and decreased disease activity in these mice, suggesting a protective role for ANXA1 on B cells, but further investigations are needed to declare this.

Interestingly, knockdown of ANXA1 both *in vivo* and *in vitro* enhanced the anti‐tumour effects of bortezomib, a commonly used treatment in multiple myeloma (MM). Cell apoptosis was enhanced in ANXA1 knockdown/bortezomib combination groups compared to that in ANXA1 knockdown alone or bortezomib‐treated alone cells, supporting a pro‐tumour role for ANXA1 in this disease [136]. Similar observations have been reported in colorectal cancer, where ANXA1 expression was associated with resistance to treatment with 5‐fluorouracil, a key drug used in the treatment of this cancer [137]. ANXA1 knockdown has also shown to suppress the proliferation, migration and invasion of non‐small‐cell lung cancer cells and has been suggested as an in vitro therapeutic target in this form of cancer [138]. To our knowledge, there are currently no ANXA1 therapies undergoing clinical trials, with most trials focusing on the use of ANXA1 as a biomarker in disease [139].

Several researchers have looked at FPR family agonists as therapeutics. An FPR1 agonist has been shown to inhibit inflammation in animal models of inflammatory ear disease [140, 141. Moreover, the inhibition of FPR1 reduced invasion and migration of lung adenocarcinoma cells during hypoxia *in vitro* [142]. There are very limited FPR1‐specific inhibitors available commercially; however, recently, two small molecule human FPR1 antagonists have been developed that selectively antagonize the function of FPR1 in human neutrophils. Researchers have suggested these antagonists seem superior to any other known antagonists; however, more detailed studies are needed with these compounds [143].

Preclinical studies have shown that FPR2 antagonists protected mice from lethal infection caused by influenza and researchers have suggested that these antagonists should be explored as new influenza treatments [144]. The FPR2 agonist Cpd43 reduced inflammation, as well as osteoclastogenesis, in a mouse model of RA [145]. Furthermore, an FPR2 agonist from Bristol‐Meyers Squibb was able to improve cardiac function in a mouse model of heart failure and promote key pro‐resolving activities such as macrophage phagocytosis [146]. This compound was also able to promote wound‐healing pathways in a mouse model of MI and preserve cardiac function after the MI [147]. The compound is currently undergoing a phase I trial in humans [148].

Other FPR2 agonists have also been investigated in humans. For instance, Actelion have conducted Phase I studies with their new FPR2 agonist, ACT‐389949, and showed that it was well tolerated; however, its therapeutic potential as an anti‐inflammatory molecule was limited [.

## CONCLUSION

The complex nature of ANXA1 and the multiple signalling pathways it is involved in perhaps contribute to the disparity in literature surrounding the molecule. This can be seen in diseases such as cancer, where the role ANXA1 plays can depend on the signalling pathways that it is involved in, as well as the localization of the molecule [47]. With a vast majority of data being sourced from animal models, it is clear that further human‐based research needs to be conducted to deeper understand the role of this protein in both health and disease before investigating its use as a potential therapeutic target or biomarker. Complementary studies investigating interactions with the FPR family in disease would be beneficial to understand the signalling pathways elicited by ANXA1, and perhaps highlight if these are dysregulated in disease.

In diseases such as arthritis or cancer, resistance to treatment is common, and in some cases side‐effects can be extreme [12, 89. Therefore, it is evident that novel treatment strategies need to be explored in these diseases. A role for ANXA1 in these diseases, and also in mediating beneficial effects of commonly used treatments such as GCs, has been implicated [11]. This provides further rationale for deeper investigation into the mechanisms of action of ANXA1 in disease, and in response to treatment with the prospect of perhaps manipulating the beneficial effects mediated by this protein to reduce treatment resistance and side‐effects. The anti‐inflammatory role of ANXA1 in innate immunity is consistent throughout the literature, in contrast to adaptive immune‐cell‐associated diseases where the part ANXA1 plays are subject to debate. ANXA1‐based therapeutics would be more beneficial initially in diseases primarily driven by innate immune cells such as atherosclerosis [8, 64, providing a basis for further research into whether this protein could perhaps also be manipulated within the adaptive immune system.

## CONFLICT OF INTEREST

Dr Fiona Dempsey and Scott Crichton are employed by the biopharmaceutical company Medannex, which is working to develop therapeutic antibodies targeting annexin‐A1.
